# Severe Anaplasmosis presenting as possible CVA: Case report and 3-year Anaplasma infection diagnosis data is based on PCR testing and serology

**DOI:** 10.1016/j.idcr.2021.e01073

**Published:** 2021-03-10

**Authors:** Yasser Eldaour, Rahman Hariri, Mohamed Yassin

**Affiliations:** a Department of Medicine, University of Pittsburgh Medical Center, Pittsburgh, PA, USA; b Division of Clinical Microbiology, University of Pittsburgh and University of Pittsburgh, Medical Center, Pittsburgh, PA, USA

**Keywords:** Anaplasma, Ticks, Seasonal variation

## Abstract

**Introduction:**

Anaplasmosis is a tick-borne illness caused by *Anaplasma phagocytophilum*. A review of CDC reports showed an increase in Anaplasmosis, with 1,193 cases reported in 2009 compared to 5,672 cases reported in 2017, with the majority of cases between May and October. Neurologic manifestations are uncommon.

**Case:**

A 72-year-old male presented in August with acute left-sided weakness. Patient was found to have an acute kidney injury (creatinine 5.3 mg/dL), thrombocytopenia (platelet count 25,000/mL), and rhabdomyolysis (CPK 25,000 units/L). Workup for an acute stroke was negative. Peripheral blood smears showed *Anaplasma* neutrophil inclusions in >30 % of the buffy coat prep. PCR testing was positive for Anaplasmosis. He was treated with doxycycline for 10 days, with improvement within 48 h. He was discharged home after a 13-day hospital course with no residual neurological deficits.

A review of our medical system between January 1st, 2016 and December 31st, 2018 revealed 20 cases of Anaplasmosis. All cases presented between May and December and had fever of unclear etiology, but only our case presented with stroke-like symptoms. All cases involved people living in heavily wooded areas, with a mean age of 70 years.

**Discussion:**

The typical presentation of Anaplasmosis is a nonspecific febrile illness with leukopenia and thrombocytopenia. Although headache is common, stroke-like symptoms are a rare but known complication. Elderly and immunocompromised patients living in heavily wooded areas are at higher risk for Anaplasmosis. Delayed diagnosis was common (55 % of case review) and associated with worse prognosis.

## Introduction

*Anaplasma phagocytophilum* is an obligate Gram-negative, intracellular bacterium that multiply within the cytoplasm of infected white blood cells. The organism is genetically related to rickettsia [5] and is transmitted by the blacklegged tick (*Ixodes scapularis*) in the northeast United States and by the western blacklegged tick (*Ixodes pacificus*) in California (west coast) [2,8,9]. The vector also transmits other organisms responsible for diseases such as Lyme, babesiosis, ehrlichiosis and Powassan encephalitis [2,8].

Anaplasmosis was formerly named human granulocytic ehrlichiosis (HGE). In 2001, the organism was reassigned to the genus *Anaplasma* [8]. Cases of anaplasmosis have been identified worldwide; in the United States, the prevalence of anaplasmosis is increasing. A review of CDC reports showed an increase in Anaplasmosis, with 1,193 cases reported in 2009 compared to 5,672 cases reported in 2017, with the majority of cases between May and October [3,1]. It is mostly reported in the upper Midwest and the Northeast. Disease activity has also been reported in Northern Europe and Southeast Asia [8].

Anaplasmosis generally presents with nonspecific symptoms such as fever, chills, malaise, headache, myalgias and occasionally non-specific rash [2]. Central nervous system involvement is uncommon in HGA, with meningoencephalitis reported in only approximately 1% of cases. In contrast, a number of different peripheral nervous system manifestations have been described, including brachial plexopathy, cranial nerve palsies, and demyelinating polyneuropathy, and bilateral facial nerve palsy, where recovery of neurologic function may be delayed over several months [10].

Co-infection with other tick-borne organisms (*Anaplasma*, Lyme, and *Babesia*) occurs given that *Ixodes*ticks are the common vector. Early signs and symptoms are nonspecific or mimic other illnesses, which can make diagnosis challenging [2]. Immunosuppressed patients are more likely to get hospitalized and have a higher risk of suffering severe complications. Death from transfusion-transmitted anaplasmosis is reported [4].

The most commonly utilized diagnostic testing for anaplasmosis are serology and PCR. PCR is the most effective diagnostic test during early-stage *A. phagocytophilum* infection with high sensitivity 74 % and specificity 100 %. Serology had a lower specificity 97 % but higher sensitivity 84 % when testing acute and convalescent samples [6,7]. The presence of intracytoplasmic aggregates of *Anaplasma* in peripheral blood neutrophils is diagnostic but only present in 20 %–80 % of symptomatic patients [8].

## Case report and 3-year review

**Fig. 1:**
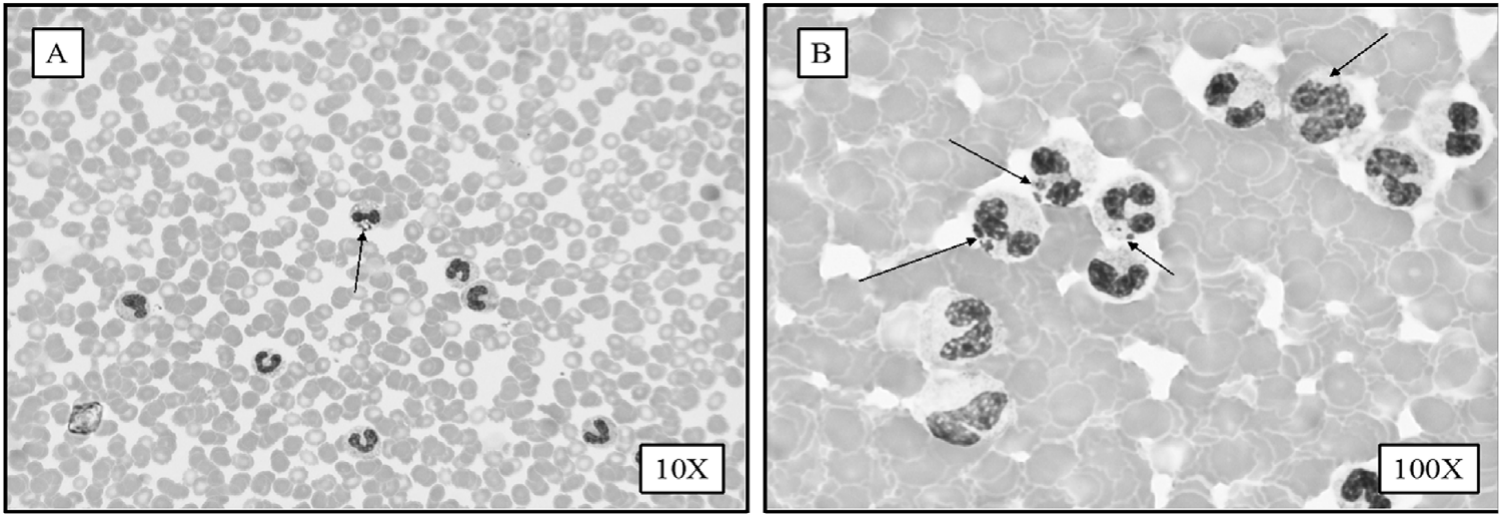
Panel A shows peripheral smear appearance at x 10 magnification. Panel B at x 100 magnification with more detailed appearance of morula within white blood cell (WBC). Panel C low power magnification (x10) showing high level of *Anaplasma* within WBCs. Panel D showing high power magnification with extensive morulae within WBCs showing severe *Anaplasma*.

A 72-year-old male with history of marginal B-Cell Lymphoma in remission presented in August with new onset left sided weakness, rhabdomyolysis, and acute injury. He was not feeling well for 3 weeks prior to presentation. Despite this, he used to cut grass in his yard and spent time in his orchard. He was found on the floor awake but confused. His lowest platelet count on admission was 25^9^/L. His creatinine was up to 5.3 mg/dL and CPK was up to 25,000 units/ L. The patient had MRI brain and was transferred to our hospital as possible cerebrovascular stroke (CVS). The MRI brain was repeated and revealed non-specific changes. The clinical evaluation of the stroke team felt that CVS is unlikely the cause of confusion. In the meantime, his peripheral smear revealed the likely diagnosis. Fig. 1 shows peripheral smears (Panel A 10X magnification and Panel B at 100X magnification of buffy coat preparation) stained with Giemsa/Wright satin shows heavy presence of multiple *Anaplasma phagocytophilum* morulae inside phagosomes of polymorphonuclear leukocytes. He was diagnosed with severe *Analplasma* infection with Neutrophil inclusions in > 30 % of the buffy coat prep. 30 %, infected PMNs on this patient was strikingly high. In most cases peripheral infected WBCc are around 2 %. Actually, diagnosis was made based on peripheral and Buffy coat smears review way before the PCR testing report became available. His *Analplasma* PCR came back positive and *Babesia and Lyme* came back negative. He was started on doxycycline with improvement within 24−48 h of antibiotic therapy. He was treated with doxycycline for 10 days and was discharged home after 13 days in hospital, fully awake with no neurological deficits.

**Fig. 2:**
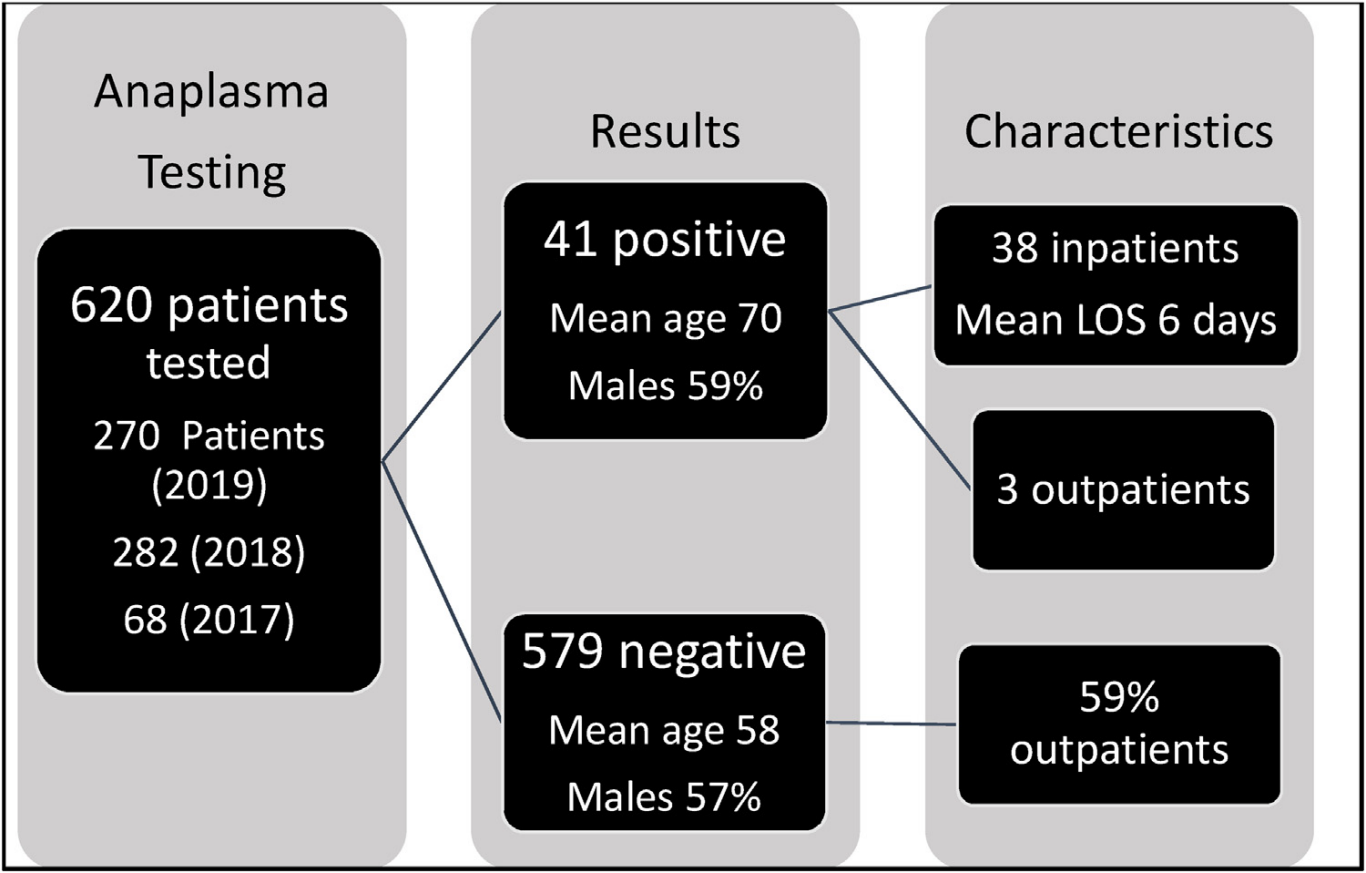
Schematic description of patients included.

We reviewed our electronic records of our medical system (17 acute care hospitals) between January 1st, 2017 and December 31st, for *Anaplasma* testing. During these three years, testing was ordered for 620 patients and revealed 41 (6.6 %) positive cases of Anaplasmosis. Fig. 2 shows schematic distribution of cases. All cases presented between May and December and had fever of unclear etiology, but only our case presented with stroke-like symptoms. The summer was the most common season with 20 (49 %) of the cases. All cases involved people living in heavily wooded areas. The delay in diagnosis is measured by days passed before testing for anaplasmosis from admission date as anaplasmosis is a community acquired infection. The delay in diagnosis was significantly affected by the month and season of the presentation. The time to testing was the shortest in summertime with a p-value of < 0.003 (CI 1.7–2.4). There was significant correlation between length of stay (LOS) and delay in diagnosis with a p-value of < 0.003 (CI 1.2–2.2).

## Discussion

The diagnosis of Anaplasmosis can be accomplished by staining of blood smears from peripheral blood, bone marrow or CSF to detect morulae. Although this method is rapid, it is relatively insensitive due to the small number of circulating cells that can be practically examined during routine microscopy, the rarity of infected cells, lack of expertise among personnel performing smear examination, and the occurrence of intracellular artifacts that may mimic morulae especially in immunocompetent patients and peripheral organs. The use of PCR-based diagnostic tests for the detection of these infections offers several advantages over the traditional serologic and blood smear tests. PCR tests have sensitivity and specificity that approach 100 %, tend to have a higher degree of sensitivity during the acute phase of illness, and have the potential to detect coinfections when configured in multiplexed reactions.

Although serology is one of major diagnostic criterion for ehrlichiosis, it has several limitations that should be considered: First, IgG IFA test is negative in as many as 80 % of patients during the first week of illness. Second, a high rate of false positive serology usually occurs due to cross reactive antigens shared by *Ehrlichia*. Third, failure to seroconvert in some cases can be attributed to immune impairment; Finally, early treatment with doxycycline occasionally reduces the antibody response to E. chaffeensis [6,7,10].

The prognosis worsens if treatment is not administered or delayed and therefore, it is important that empiric therapy with doxycycline be started for any patient with compatible clinical and laboratory findings. Initial diagnosis of ehrlichiosis can be based on nonspecific biochemical and hematological findings. However, confirmatory tests should be performed at different intervals after the onset of illness.

## Author statement

The manuscript was written by the first author Dr. Eldaour. Dr. Hariri performed the blood smear, identified Anaplasma, took the pictures and reviewed manuscript. Dr. Yassin provided the 2- year review of cases, reviewed the manuscript and provided response to the reviewers.

## Declaration of Competing Interest

The authors declare that they have no conflict of interest. All authors contribute to the manuscript submission. YE wrote the manuscript, RH performed laboratory diagnostics and obtained pictures and MY obtained patients’ data and reviewed manuscript.
